# ST-segment elevation myocardial infarction: Historical perspective and new horizons

**DOI:** 10.1007/s12471-020-01443-9

**Published:** 2020-08-11

**Authors:** F. Zijlstra, H. Suryapranata, M.-J. de Boer

**Affiliations:** 1 Department of Cardiology, Erasmus MC, University Medical Center Rotterdam, Rotterdam, The Netherlands; 2 Department of Cardiology, Radboud University Medical Center Nijmegen, Nijmegen, The Netherlands

**Keywords:** ST elevation myocardial infarction, History, Future perspectives, Primary angioplasty, OHCA

## Abstract

After a brief history of the emergence of modern therapy for acute ST-elevation myocardial infarction, we discuss the issues that dominate ongoing studies and are the focus of intense debates. The role of angiography, pharmacotherapy, thrombus aspiration, management of multi-vessel disease, mechanical complications and cardiogenic shock and the quest for myocardial salvage are discussed.

## Dutch contribution to the field

- The Zwolle Myocardial Infarction studies
- The Zwolle Primary Angioplasty trials
- The Coronary Angiography after Cardiac Arrest (COACT) trial

## Introduction

Acute myocardial infarction presenting with sustained ST-segment elevation is thought to be the result of the sudden and complete obstruction of a coronary artery by the formation of a thrombus at the site of a fissured or ruptured atherosclerotic plaque [1, 2]. In the 1960s and 1970s management consisted of identifying and treating ventricular arrhythmias and other complications. In the late 1970s Andreas Gruntzig and Peter Rentrop described new therapies that could be used to achieve reperfusion in patients presenting with acute myocardial infarction [3, 4]. Intracoronary and intravenous thrombolytic therapy and percutaneous transluminal coronary balloon angioplasty, alone or in combination, could successfully be applied to restore antegrade flow in the epicardial coronary artery [3–6]. Following randomised comparisons, it gradually became apparent that primary angioplasty resulted in better clinical outcomes compared with thrombolysis and was shown to be applicable on a nationwide scale [7–9]. It has also become clear that time delay to reperfusion does count, not only for thrombolysis, but also for primary angioplasty [10]. Prehospital triage has been generally introduced in the developed world and has a major positive impact on delays to reperfusion and total ischaemic time [11, 12]. After initial restoration of antegrade flow by means of wire, balloon or thrombus aspiration, stenting with a modern drug-eluting stent has become generally accepted [13–15].

## The pivotal role of acute coronary angiography

The safety and diagnostic potential of coronary angiography during the early hours of acute myocardial infarction was reported 40 years ago [2]. A radial procedure is generally preferred, except in case of cardiogenic shock with a need for mechanical support. In addition to being a prelude to percutaneous coronary intervention (PCI) of the infarct-related coronary artery, acute coronary angiography allows identification of patients with multi-vessel disease who may need additional revascularisation of non-infarct-related arteries. Furthermore, clinical scenarios without thrombotic coronary obstruction such as Takotsubo syndrome and spontaneous coronary artery dissections or myocardial infarctions due to dissections of the aortic root and other conditions that may require surgical intervention can be identified and treated accordingly [16].

## Pharmacotherapy

According to current guidelines [17], to prevent stent thrombosis and/or recurrent myocardial infarction all patients are treated with dual antiplatelet therapy consisting of aspirin in combination with either clopidogrel, prasugrel or ticagrelor, usually for one year. Other durations, in particular shorter, as well as how to deal with patients with atrial fibrillation necessitating anticoagulant therapy are currently being studied. A tailored approach based on the balance of bleeding versus thrombotic risks may become the best option [18]. Intravenous heparin is essential during acute PCI to prevent catheter thrombosis. A large number of additional pharmacological interventions have been studied to further improve clinical outcomes. New advances in antithrombotic therapy together with preventive measures after ST-segment elevation myocardial infarction (STEMI) have been studied extensively as the clinical syndrome is an acute thrombus-driven event. Oral antiplatelet agents such as aspirin and P2Y12 inhibitors like prasugrel, ticagrelor and intravenous antiplatelet agents (abciximab, eptifibatide and tirofiban), and intravenous anticoagulant agents (unfractionated heparin, low-molecular-weight heparin and bivalirudin) are the focus of research. Recently it was suggested that prasugrel might be more effective than other antiplatelet agents, without an increased bleeding risk [19]. Furthermore, cangrelor, a rapid onset and potent intravenous P2Y12 inhibitor, became available but its role has yet to be determined [20]. A personalised approach using genetic testing to adjust and guide antiplatelet therapy may further improve outcome especially in high-risk patients [21]. Many antithrombotic regimens, gluco-metabolic interventions and a host of other pharmacological interventions have been studied, often with promising evidence in pre-clinical studies, but so far without consistent positive results in clinical settings. As preprocedural TIMI flow, before angioplasty, is a major determinant of survival, there is a need for pharmacological interventions, including thrombolytic therapy, either at home or in the ambulance, before cath-lab arrival. Optimal secondary prevention and rehabilitation are important for long-term outcome [17].

## Thrombus aspiration

The importance of the thrombotic component of acute coronary obstruction presenting as STEMI was recognised from the early days of angiography-guided therapy of myocardial infarction [1, 2], and embolisation is a fundamental component of the pathophysiology of myocardial infarction [22]. If there is angiographic evidence of embolisation the prognosis is worse [23], and removal of (part of) the thrombus seems a rational option, already described in the first report of systematic angiography in patients with myocardial infarction [2]. Following positive initial results [24], large randomised studies [25, 26] did not demonstrate consistent clinical benefits, and the current guidelines do not advocate systematic application but leave some room for selective use based on angiographic signs of thrombus load [17]. However, an individual pooled meta-analysis of trials including >1000 patients comparing primary PCI with and without thrombus aspiration did not identify specific subgroups that may benefit from this approach [27]. A potential explanation is the marked operator dependence of outcomes after primary PCI, in particular described after thrombus aspiration [28]. Unfortunately, most public reporting and research of clinical outcomes of patients treated with primary PCI as well as PCI for stable coronary artery disease do not include the operator as baseline variable.

## How to manage patients with multi-vessel disease?

About half of the patients presenting with acute STEMI have significant multi-vessel coronary artery disease on initial coronary angiography. Whether to aim for complete revascularisation, either acute, (semi)elective or after non-invasive evidence of residual myocardial ischaemia, is an important strategic consideration with recent evidence tilting towards complete revascularisation either prior to discharge or early after hospital discharge [29–34]. Complete revascularisation in selected patients with multi-vessel disease during the initial primary PCI is safe and cost-effective in haemodynamically stable patients but cannot be recommended in those presenting with cardiogenic shock [35]. In haemodynamically stable patients, there seems to be a role for systematic use of fractional flow reserve (FFR) guidance, as functional lesion severity of non-culprit lesions may often be unclear. Further studies comparing acute-complete with staged-complete FFR-guided revascularisation are currently ongoing. An interesting alternative approach after initial treatment of the culprit lesion that should be investigated is early (within days) non-invasive testing with MRI [36]. The current evidence suggests that there will be no ‘one-size-fits-all’ solution, and we need to work towards a patient-tailored strategy taking into account in particular haemodynamic status and lesion complexity.

## Mechanical complications and cardiogenic shock

Clinical outcomes in STEMI have improved markedly over the last four decades, but there remain two clinical scenarios where we have remained unsuccessful in our attempts to improve prognosis: mechanical complications and cardiogenic shock. Mechanical complications have become uncommon following the introduction of reperfusion therapy, in particular primary PCI, but when they occur, they are still associated with a high mortality in particular without appropriate surgical intervention. These devastating complications of myocardial infarction include free wall rupture, ventricular septal defect and papillary muscle rupture. A US hospital database covering the years 2003–2015 showed an incidence of 10,726 patients with a mechanical complication from 3,951,861 STEMIs (0.27%) with an in-hospital mortality rate of 42.4%. The incidence and outcomes did not change over time. A major predictor of poor outcome in this large cohort was cardiogenic shock at presentation [37]. Unfortunately, we have made very little progress in the management of cardiogenic shock in patients with STEMI. Complete revascularisation has not improved outcome and mechanical support by means of an intra-aortic balloon pump has not been shown to work as bridge to recovery [35, 38]. Although there is some evidence that very early (within 1–2 h after symptom onset) primary PCI may prevent the development of cardiogenic shock and establish reperfusion within a time frame that allows rapid restoration of left ventricular function, new developments in mechanical support and interventions aimed at myocardial salvage are urgently needed [39, 40].

## The quest for myocardial salvage

The restoration of antegrade flow in the epicardial coronary artery in patients treated with primary PCI results, in most patients, in a rapid decrease in symptoms, normalisation of ST‑T segment abnormalities on the 12-lead electrocardiogram, limited impact on left ventricular function and good clinical outcome. However, poor myocardial reperfusion as documented by an absent or poor myocardial blush on angiography and sustained ST elevation is a clear sign of extensive irreversible damage. Although the clinical correlates are well described, the fundamental pathophysiology is poorly understood [23, 41, 42]. Distal embolisation no-reflow, reperfusion injury and probably several other as yet undefined mechanisms form the next barrier to further improve outcomes of primary PCI, in particular in patients with cardiogenic shock, and to prevent late progression to heart failure. Intracoronary antiplatelet agents, vasodilators such as adenosine, post-conditioning with 3–4 balloon inflations, thrombus aspiration, proximal as well as distal embolic protection, catheter-based cell therapy, systemic therapies with beta-blockers, cyclosporine, glucose-insulin-potassium, erythropoietin, intravenous cell therapy, remote pre- and post-conditioning and various other interventions have all been studied in small or medium sized pre-clinical and clinical trials with often encouraging biological signals in pre-clinical conditions, but so far without any impact on clinical practice.

## Out-of-hospital cardiac arrest and STEMI

Although no benefit of an early angiography approach in patients presenting with out-of-hospital cardiac arrest (OHCA) without ST elevation has been demonstrated [43], those presenting *with* ST elevation are a subgroup of special interest as these patients may especially benefit from an early invasive strategy combined with optimal prehospital logistics. They seem to be vulnerable for ventricular arrhythmias, sometimes with only small culprit vessels involved, and there is strong evidence that genetic modulators are responsible for this often lethal combination [44]. Early identification and treatment of these patients may be an important step to further improve prognosis of STEMI-related sudden cardiac arrest. Increased awareness and performance of bystander cardiopulmonary resuscitation (CPR) and national and regional initiatives to improve lay person CPR together with 24/7 automated external defibrillator public accessibility may contribute to improving outcome after STEMI with sudden cardiac arrest [45].

**Fig. 1:**
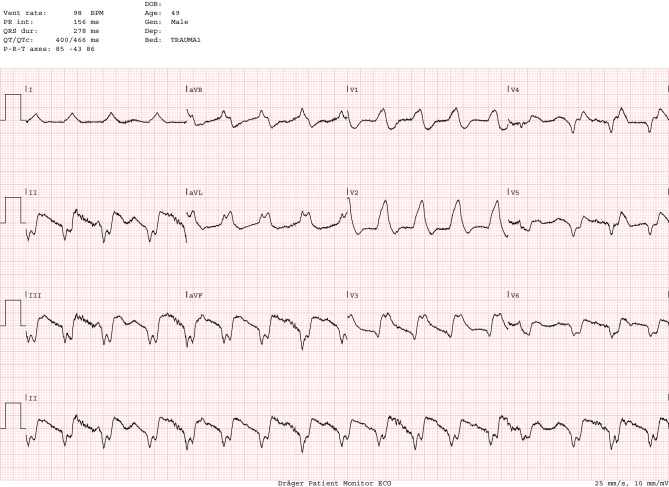
ECG on admission

**Fig. 2:**
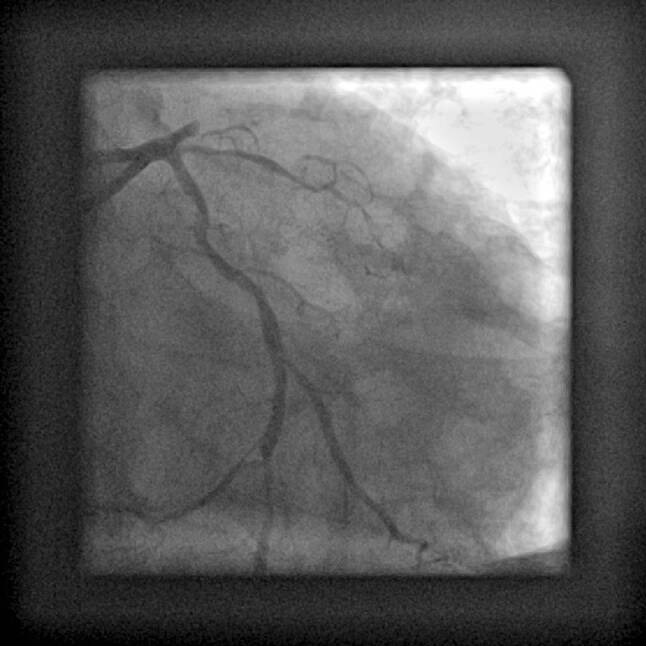
Acute angiography of the left coronary artery showing an acute thrombotic occlusion of the proximal left anterior descending artery (LAD) and significant stenosis in the circumflex artery

**Fig. 3:**
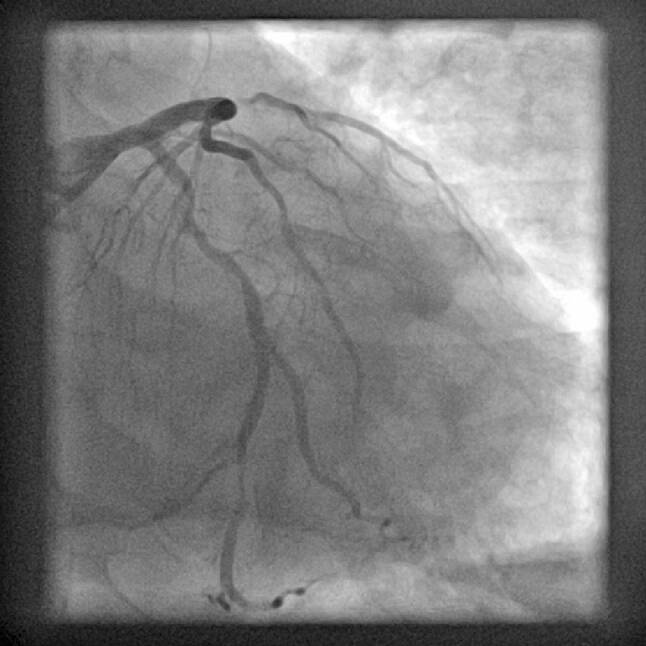
The final result after primary angioplasty with good antegrade filling of the LAD including the diagonal and septal branches

To illustrate several of the discussed issues, we conclude with a case: A 49-year-old male was admitted after a witnessed out-of-hospital cardiac arrest, with an estimated delay of 8 min to basic life support, initial rhythm ventricular fibrillation. Refractory ventricular fibrillation and cardiogenic shock necessitated mechanical ventilation and after arrival in the emergency room venoarterial extracorporeal membrane oxygenation (V‑A ECMO) was instituted percutaneously by the femoral route and running 63 min after start of the resuscitation. The 12-lead electrocardiogram is shown in Fig. 1. Coronary angiography through the right radial artery showed a chronic total occlusion of the proximal right coronary artery and an acute thrombotic occlusion of the left anterior descending coronary artery with several diffuse intermediate lesions in the circumflex artery (Fig. 2). After predilatation with a 2 mm balloon, two (4/22 and 3/18 mm) overlapping drug-eluting stents were placed with restoration of TIMI 3 flow (Fig. 3) resulting in sustained sinus rhythm, normalisation of QRS width and ST-elevation resolution. Despite marked elevations of cardiac markers, the haemodynamic situation stabilised rapidly, and after 24 h of moderate hypothermia, sedation was stopped. The patient showed full neurological recovery and mechanical ventilation could be stopped on day 2. Based on daily echocardiography with reduced V‑A ECMO flow, this mechanical support was continued to day 4, then weaned and explanted. Clinically the patient did well, with a left ventricular ejection fraction on day 5 of 35%. An FFR-guided PCI of the circumflex artery was scheduled prior to discharge.
